# Adult attachment profiles, death attitudes, and intention to remain in nursing among Chinese intern nursing students

**DOI:** 10.3389/fmed.2026.1803579

**Published:** 2026-06-29

**Authors:** Yan Yang, Jing Hua, Chenghao Shi, Chenling Zhu, Yuping Zhang, Danni Lin, Fang Chen

**Affiliations:** Department of Nursing, The Second Affiliated Hospital Zhejiang University School of Medicine, Hangzhou, China

**Keywords:** adult attachment, death attitude, intention to remain in nursing, latent profile analysis, nursing students, nursing education

## Abstract

**Background:**

Intern nursing students are frequently exposed to patient death during clinical training, yet many receive limited preparation for coping with death-related experiences. Adult attachment may shape how students regulate distress and respond to death-related stressors, but little is known about attachment profiles and their associations with death attitudes and intention to remain in nursing.

**Aims:**

This study aimed to identify latent profiles of adult attachment among Chinese intern nursing students and to examine their associations with death attitudes and intention to remain in nursing.

**Setting and participants:**

A cross-sectional study was conducted in three teaching hospitals in Hangzhou, China. A total of 1,124 intern nursing students were recruited via convenience sampling.

**Methods:**

Data were collected through an online questionnaire. The Adult Attachment Questionnaire, 10-item short form, the Death Attitude Profile–Revised, and a single-item measure of intention to remain in nursing were administered. Latent profile analysis was performed to identify attachment profiles. Profile differences in death attitudes were examined using ANOVA and ANCOVA adjusted for education level. Associations with intention to remain in nursing were assessed using chi-square tests and adjusted logistic regression.

**Results:**

Four distinct attachment profiles were identified: “relatively secure” (41.6%), “mildly insecure” (29.4%), “moderately insecure” (16.5%), and “highly insecure” (12.5%). Profile differences were significant across all five DAP-R subscales and remained significant after adjustment for education level. Compared with the relatively secure profile, the highly and moderately insecure profiles showed higher fear of death, death avoidance, and escape acceptance, and lower neutral acceptance. In adjusted logistic regression, the highly insecure profile (OR = 0.348, 95% CI [0.228, 0.531], *p* < 0.001) and moderately insecure profile (OR = 0.563, 95% CI [0.373, 0.850], *p* = 0.006) had lower odds of intending to remain in nursing.

**Conclusion:**

Distinct adult attachment profiles were associated with death attitudes and intention to remain in nursing among Chinese intern nursing students. Students with higher attachment insecurity showed less favorable death-attitude patterns and lower intention to remain in nursing. These findings may inform targeted death education and psychological support, but should be interpreted cautiously given the cross-sectional design, self-reported data, and single-item outcome measure.

## 1 Introduction

According to global nursing workforce shortages, intern nursing students constitute a critical future healthcare resource; their intention to remain in nursing is therefore a key concern for health system workforce sustainability. Intern nursing students are at a pivotal transitional stage between academic training and independent professional practice, during which they are first systematically exposed to patient death and dying in real clinical settings. During the internship period, frequent encounters with death, combined with heavy workloads and limited clinical experience, place substantial emotional and psychological demands on interns (1–3), which may have implications for their intention to remain in nursing (1, 4).

Negative attitudes toward death are clinically important because they have been linked to worse psychological wellbeing, poorer end-of-life care engagement, and a stronger intention to leave the nursing profession (5, 6). Death attitudes are commonly conceptualized as a multidimensional construct encompassing fear of death, death avoidance, and different forms of acceptance, as assessed by the Death Attitude Profile-Revised (DAP-R) (4, 7). Although death education has been reported to improve death attitudes, students’ responses vary substantially, suggesting that individual differences may shape how interns appraise and cope with patient death (8–10).

Adult attachment theory offers a robust framework for understanding individual differences in emotion regulation, threat appraisal, and coping in high-stress interpersonal contexts (11). Attachment is typically conceptualized along two dimensions: anxiety and avoidance (12). Higher levels of attachment insecurity have been associated with greater death anxiety, maladaptive emotional responses to patient death, increased burnout, and lower professional commitment among nurses and medical trainees (13–16).

However, prior research has largely used variable-centered approaches and has focused on general adult populations or university students, with limited evidence in intern nursing students (17, 18). As a result, it remains unclear whether distinct attachment patterns exist in this group and how such patterns relate to death attitudes and intention to remain in nursing during clinical training.

Latent profile analysis (LPA) is a person-centered approach that can address this heterogeneity by identifying distinct subgroups within a population based on characteristic patterns across multiple variables, specifically attachment anxiety and avoidance (19, 20). Unlike variable-centered approaches that examine attachment anxiety and avoidance as separate continuous predictors, LPA identifies co-occurring combinations of these dimensions, enabling recognition of distinct subgroups that may differ meaningfully in death attitudes and intention to remain in nursing (21). Therefore, the present study employed latent profile analysis to identify distinct profiles of adult attachment among intern nursing students in China and to examine the associations between these profiles and death attitudes and intention to remain in nursing.

## 2 Materials and methods

### 2.1 Study design

This study used a cross-sectional design. We report the study in accordance with the Strengthening the Reporting of Observational Studies in Epidemiology (STROBE) statement.

### 2.2 Participants

The participants were recruited via convenience sampling from three teaching hospitals in Hangzhou, Zhejiang province, China. The inclusion criteria were as follows: (1) being enrolled in an accredited pre-licensure nursing program and currently undertaking a hospital-based clinical internship (clinical placement), (2) not yet holding a registered nurse license at the time of the survey, and (3) providing complete responses to the study instruments. Previous studies on latent profile analysis have shown that, when profiles are well-separated, and the smallest profile comprises at least about 10% of the sample, a total sample size of 300–500 is generally sufficient to obtain stable solutions for models (21).

### 2.3 Data collection

An online questionnaire was developed using the Wenjuanxing platform^1^, converted into a QR code, and distributed to participants across three teaching hospitals. To minimize item-level missing data, all questionnaire items were set as mandatory before submission. The overall questionnaire took 10–15 min to complete.

### 2.4 Measurement

Demographic characteristics: Data concerning basic socio-demographic characteristics were collected, including age, gender, education level, place of birth, and whether they have a plan to continue applying for further education.

The adult attachment questionnaire (AAQ): The AAQ was used to assess individuals’ feelings about close relationships (22). Du and colleagues translated and psychometrically revised the AAQ and derived a validated 10-item short form (AAQ-10) in Chinese college student samples (23). In this study, we used the AAQ-10, which comprises two dimensions: attachment avoidance (five items) and attachment anxiety (five items). Items are rated on a seven-point Likert scale (1 = strongly disagree to 7 = strongly agree). This scoring format follows the validated Chinese version of the AAQ-10. Subscale scores were computed by summing the corresponding items (range: 5–35), with higher scores indicating greater attachment avoidance or attachment anxiety (i.e., higher attachment insecurity). In the present study, Cronbach’s α for avoidance and anxiety were 0.92 and 0.88, respectively.

The Death Attitude Profile-Revised (DAP-R): The DAP-R assesses attitudes toward death and includes 32 items across five subscales: fear of death (seven items), death avoidance (five items), neutral acceptance (five items), approach acceptance (10 items), and escape acceptance (five items). Items are rated on a five-point Likert scale (1 = strongly disagree to 5 = strongly agree). The Chinese version shows good internal consistency (Cronbach’s α = 0.875) (24), and Cronbach’s α of each domain in this study were 0.83, 0.76, 0.75, 0.86, and 0.87.

Intention to remain in nursing: Intention to remain in nursing was defined as an intern’s self-reported intention to pursue or remain in the nursing profession. It was measured with a single yes/no item in the current study: “*Do you intend to work as a nurse after your current internship*?” and treated as a binary variable in the analyses. Although intention to remain in nursing was assessed using a single-item measure, similar approaches have been widely used in prior studies, including dichotomous (yes/no) measures of intention to stay or leave the profession (25). This item was used as a brief indicator of students’ current intention to remain in nursing, rather than as a comprehensive measure of professional commitment or actual retention behavior.

### 2.5 Ethical considerations

The Ethics Committee of the Second Affiliated Hospital of Zhejiang University School of Medicine approved the study (approval number: 2024-0503). This study was conducted in accordance with the Declaration of Helsinki. The participants were informed of their voluntary and anonymous participation. Each participant was required to complete an electronic informed consent form before proceeding with the questionnaire. Completion and submission of the questionnaire were regarded as consent to participate. No financial compensation or incentives were provided for participation.

### 2.6 Statistical analyses

The statistical analyses were conducted using SPSS 26.0 and Mplus 8.3. Descriptive analyses were used to summarize the socio-demographic variables of the participants. Group differences in categorical variables were examined using chi-squared or Fisher’s exact tests, and continuous variables were examined using independent-samples *t*-tests or one-way ANOVA; a two-sided *p* < 0.05 was considered statistically significant.

Latent profile analysis (LPA) of adult attachment was conducted in Mplus 8.3 using attachment avoidance and attachment anxiety as continuous indicators. Models with one to six profiles were fitted, and the optimal number of profiles was determined by jointly considering the Akaike Information Criterion (AIC), Bayesian Information Criterion (BIC), sample-size adjusted Bayesian Information Criterion (aBIC), entropy, Lo-Mendell-Rubin likelihood ratio test (LMR), and bootstrap likelihood ratio test (BLRT). Lower values of AIC, BIC, and aBIC indicate a better fit, while significant results (*p* < 0.05) from LMR and BLRT suggest that the k-profile model outperformed the k-1 profile model. Classification accuracy was assessed using the entropy index, with values above 0.8 indicating high classification accuracy. To avoid local maxima, multiple sets of random starting values were employed in Mplus; the optimal solution was accepted only when the best loglikelihood value was successfully replicated. Average posterior probabilities of class membership were also computed to evaluate profile-specific classification accuracy.

After the optimal profile solution was selected, profile membership was exported to SPSS 26.0. Preliminary analyses were conducted to examine whether demographic characteristics differed across attachment profiles. As education level differed significantly across profiles and has been previously associated with death attitudes, it was treated as a covariate in subsequent adjusted analyses. Death attitude scores were summarized as means and standard deviations across attachment profiles. Differences in death attitude scores were first examined using one-way ANOVA with Bonferroni-corrected *post hoc* comparisons. ANCOVA models were additionally conducted with education level as a covariate. Adjusted estimated marginal means (EMMs) with 95% confidence intervals were estimated using the relatively secure profile as the reference group. The association between attachment profiles and intention to remain in nursing (yes/no) was assessed using chi-squared tests and adjusted logistic regression models controlling for education level (26, 27).

### 2.7 Common method bias test

As all variables were collected through self-reported surveys at a single time point, common method bias and social desirability bias were considered. To mitigate these risks, procedural remedies were applied, including anonymous data collection and standardized measurement procedures. In addition, Harman’s single-factor test showed that the first factor accounted for 24.307% of the total variance, below the recommended threshold, suggesting that common method bias was unlikely to substantially affect the results.

## 3 Results

### 3.1 Descriptive statistics

Overall, 1,196 participants completed the questionnaires, and 1,124 responses were included in the analysis (effective response rate: 94.0%). The mean age of the sample was 21.97 years, 88.2% were female, and 79.1% of participants reported that they intended to remain in nursing (Table 1). Preliminary analyses (Table 1) revealed that gender (*χ^2^* = 1.737, *p* = 0.629), age (*F* = 0.951, *p* = 0.415), place of origin (*χ^2^* = 2.599, *p* = 0.458), and education plan (*χ^2^* = 0.875, *p* = 0.831) did not significantly differ across attachment profiles. However, education level showed significant variation across profiles (*χ^2^* = 14.124, *p* = 0.028). Therefore, education level was included as a covariate in subsequent adjusted analyses.

**TABLE 1:** A univariate analysis of potential types of adult attachment among intern nursing students.

Characteristic	*n* (%)	Adult attachment				Value	*P*
		Moderately insecure attachment profile (Class 1)	Highly insecure attachment profile (Class 2)	Relatively secure attachment profile (Class 3)	Mildly insecure attachment profile (Class 4)		
		*n* = 186, 16.5%	*n* = 140, 12.5%	*n* = 468, 41.6%	*n* = 330, 29.4%		
Age (M ± SD)	21.97 ± 1.31	21.97 ± 1.26	22.06 ± 1.16	22.01 ± 1.48	21.87 ± 1.13	0.951^a^	0.415
Gender						1.737^b^	0.629
Female	991 (88.2)	166 (89.2)	122 (87.1)	407 (87.0)	296 (89.7)	–	–
Male	133 (11.8)	20 (10.8)	18 (12.9)	61 (13.0)	34 (10.3)	–	–
Place of born						2.599^b^	0.458
Zhejiang	670 (59.6)	115 (61.8)	87 (62.1)	266 (56.8)	202 (61.2)	–	–
Outside of Zhejiang	454 (40.4)	71 (38.2)	53 (37.9)	202 (43.2)	128 (38.8)	–	–
Education level						14.124^b^	**0.028**
Associate degree	52 (4.6)	13 (7.0)	8 (5.7)	24 (5.1)	7 (2.1)	–	–
Bachelor degree	978 (87.0)	157 (84.4)	125 (89.3)	395 (84.4)	301 (91.2)	–	–
Master degree	94 (8.4)	16 (8.6)	7 (5.0)	49 (10.5)	22 (6.7)	–	–
Intention to remain in nursing						30.745^b^	**<0.001**
Yes	889 (79.1)	138 (74.2)	89 (63.6)	392 (83.8)	270 (81.8)	–	–
No	235 (20.9)	48 (25.8)	51 (36.4)	76 (16.2)	60 (18.2)	–	–
Plan to pursue further education	–	–	–	–	–	0.875^b^	0.831
Yes	700 (62.3)	113 (60.8)	90 (64.3)	296 (63.2)	201 (60.9)	–	–
No	424 (37.7)	73 (39.2)	50 (35.7)	172 (36.8)	129 (39.1)	–	–

M, mean; SD, standard deviation.

^a^One-way analysis of variance.

^b^Chi-square (χ^2^) test. Bold values indicate statistical significance (*p* < 0.05).

Comparisons across education levels revealed significant differences in attachment anxiety, fear of death, neutral acceptance, approach acceptance, and escape acceptance (all *p* < 0.05), full results are presented in Supplementary Table 1.

### 3.2 Latent profile model selection

Latent profile models with one to six profiles were estimated, and model fit was evaluated using AIC, BIC, aBIC, entropy, LMR, and BLRT (Table 2). Although information criteria continued to decrease with additional profiles, the five- and six-profile solutions were not retained. Specifically, the best loglikelihood values for these models were not replicated across random starting values, indicating unstable solutions. The six-profile solution included one profile comprising less than 10% of the total sample (*n* = 51), and both the five- and six-profile solutions yielded profiles that were conceptually indistinguishable from adjacent profiles, suggesting over-extraction. The four-profile solution was selected as the optimal solution based on converging statistical and theoretical criteria: it demonstrated high classification accuracy (entropy = 0.956), average posterior probabilities of class membership ranged from 0.928 to 0.998, and significant LMR and BLRT results relative to the three-profile model (*p* < 0.001). The four profiles were furthermore theoretically interpretable as a meaningful gradient of attachment security, each sufficiently large for stable estimation (minimum profile size: 12.5%).

**TABLE 2:** Overview of model fit indices of latent profile analysis (*n* = 1124).

Classes	k	AIC	BIC	aBIC	Entropy	LMR value	LMR *p*	BLRT *p*	Class proportion
1	4	14365.448	14385.546	14372.841	−	−	−	−	−
2	7	13942.391	13977.564	13955.330	0.840	409.619	<0.001	<0.001	0.707/0.293
3	10	13712.224	13762.470	13730.707	0.890	225.469	<0.001	<0.001	0.444/0.225/0.331
4	13	13275.342	13340.662	13299.370	0.956	422.818	<0.001	<0.001	0.165/0.125/0.416/0.294
5	16	13141.409	13221.803	13170.983	0.958	133.593	<0.001	<0.001	0.416/0.254/0.102/0.123/ 0.104
6	19	13058.723	13154.191	13093.842	0.960	84.668	<0.001	<0.001	0.109/0.076/0.416/0.045/ 0.100/0.254

AIC, Akaike Information Criterion; BIC, Bayesian Information Criterion; aBIC, sample-size adjusted BIC; LMR, Lo-Mendell-Rubin likelihood ratio test; BLRT, bootstrap likelihood ratio test. Lower AIC, BIC, and aBIC values indicate better fit; significant LMR and BLRT (*p* < 0.05) indicate that the k-profile model fits better than the k−1 profile model. “−”, not applicable for the 1-profile model.

### 3.3 Characteristics of each profile

**FIGURE 1:**
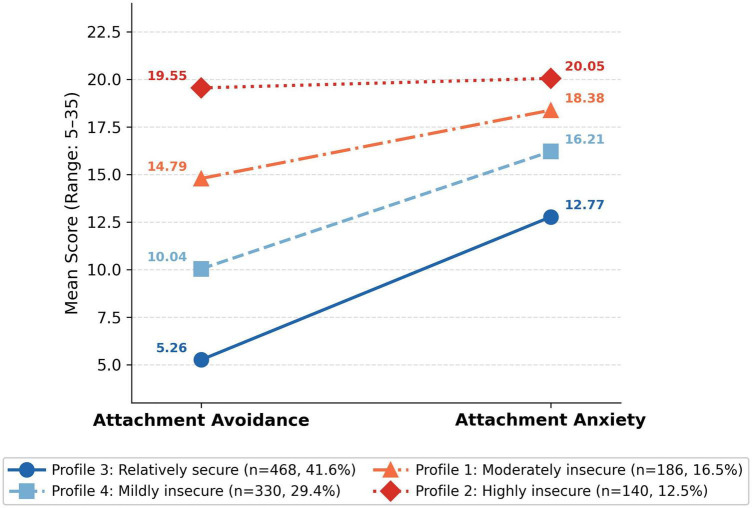
Mean attachment avoidance and anxiety scores across the four adult attachment profiles (*N* = 1,124).

Table 3 presents the profile-specific means for attachment avoidance and attachment anxiety, as well as the number and proportion of participants in each profile, for the retained four-profile solution identified by latent profile analysis. Overall, a gradient pattern was observed across profiles. Profile 1 comprised 186 participants (16.5%) and showed elevated levels of both avoidance (14.79) and anxiety (18.38), and was labeled the moderately insecure attachment profile. Profile 2, the smallest profile (*n* = 140, 12.5%), exhibited the highest mean levels of avoidance (19.55) and anxiety (20.05) and was labeled the highly insecure attachment profile. Profile 3 was the largest profile (*n* = 468, 41.6%) and showed the lowest mean scores on avoidance (5.26) and anxiety (12.77), corresponding to the relatively secure attachment profile. Profile 4 comprised 330 participants (29.4%) and showed intermediate scores on avoidance (10.04) and anxiety (16.21), and was labeled the mildly insecure attachment profile. This gradient pattern from lowest to highest insecurity across Profiles 3, 4, 1, and 2 is shown in Figure 1.

**TABLE 3:** Latent profiles of adult attachment among intern nursing students (*N* = 1,124).

Attachment profile	*n*	%	Attachment avoidance, M ± SD	Attachment anxiety, M ± SD
Profile 1 moderately insecure	186	16.5	14.79 ± 1.29	18.38 ± 6.20
Profile 2 highly insecure	140	12.5	19.55 ± 1.00	20.05 ± 5.14
Profile 3 relatively secure	468	41.6	5.26 ± 0.59	12.77 ± 6.79
Profile 4 mildly insecure	330	29.4	10.04 ± 1.11	16.21 ± 6.31

Avoidance and anxiety were measured by the Adult Attachment Questionnaire, 10-item short form (AAQ-10); higher scores indicate greater attachment avoidance or anxiety.

### 3.4 Associations between latent profiles, death attitudes and intention to remain in nursing

Intention to remain in nursing differed significantly across profiles (*χ^2^* = 30.745, *p* < 0.001; Table 4). The highly insecure profile (Profile 2) had the lowest proportion intending to remain (63.6%), compared with 83.8% in the relatively secure profile (Profile 3) and 81.8% in the mildly insecure profile (Profile 4).

**TABLE 4:** Intention to remain in nursing by adult attachment profile (*N* = 1,124).

Attachment profile	Intend to remain in nursing, *n* (%)	Do not intend to remain in nursing, *n* (%)
Profile 1 moderately insecure	138 (74.2)	48 (25.8)
Profile 2 highly insecure	89 (63.6)	51 (36.4)
Profile 3 relatively secure	392 (83.8)	76 (16.2)
Profile 4 mildly insecure	270 (81.8)	60 (18.2)
Total	889 (79.1)	235 (20.9)

χ^2^ = 30.745, *p* < 0.001.

In adjusted logistic regression analyses with education level as a covariate and the relatively secure profile as the reference group, both the highly insecure profile (OR = 0.348, 95% CI [0.228, 0.531], *p* < 0.001) and the moderately insecure profile (OR = 0.563, 95% CI [0.373, 0.850], *p* = 0.006) were associated with significantly lower odds of intending to remain in nursing. The mildly insecure profile did not differ significantly from the relatively secure profile (OR = 0.887, 95% CI [0.610, 1.289], *p* = 0.529).

Unadjusted descriptive statistics are presented in Table 5; ANCOVA-adjusted estimated marginal means (EMM) with 95% confidence intervals, controlling for education level, are presented in Table 6. Profile differences were observed across all five DAP-R subscales.

**TABLE 5:** Death attitudes (DAP-R scores) by adult attachment profile among intern nursing students (unadjusted).

Item	Adult attachment profile, score M ± SD					ANOVA test	
	Overall	Moderately insecure (Profile 1)	Highly insecure (Profile 2)	Relatively secure (Profile 3)	Mildly insecure (Profile 4)	*F*	*P*
	*n* = 1,124	*n* = 186 (16.5%)	*n* = 140 (12.5%)	*n* = 468 (41.6%)	*n* = 330 (29.4%)		
Fear of death	2.47 ± 0.70	2.66 ± 0.68	2.70 ± 0.61	2.26 ± 0.72	2.57 ± 0.64	27.996	**<0.001**
Death avoidance	2.74 ± 0.73	2.84 ± 0.63	2.82 ± 0.57	2.66 ± 0.83	2.76 ± 0.66	3.876	**0.009**
Neutral acceptance	4.17 ± 0.53	4.05 ± 0.52	3.86 ± 0.62	4.34 ± 0.47	4.12 ± 0.48	40.640	**<0.001**
Approach acceptance	2.54 ± 0.65	2.70 ± 0.54	2.74 ± 0.59	2.36 ± 0.73	2.62 ± 0.55	22.739	**<0.001**
Escape acceptance	2.24 ± 0.80	2.56 ± 0.71	2.65 ± 0.70	1.96 ± 0.81	2.30 ± 0.71	47.545	**<0.001**

Unadjusted means (M ± SD) are presented for descriptive purposes. ANCOVA-adjusted estimated marginal means with 95% confidence intervals are provided in Table 6. Bold values indicate statistical significance (*p* < 0.05).

**TABLE 6:** ANCOVA-adjusted estimated marginal means of DAP-R subscale scores by adult attachment profile (covariate: education level).

Item	Moderately insecure (Profile 1) M [95% CI]	Highly insecure (Profile 2) M [95% CI]	Relatively secure (Profile 3) M [95% CI]	Mildly insecure (Profile 4) M [95% CI]	Adjusted *F*	*P*	*Post hoc* (Bonferroni)
Fear of death	2.66 [2.56, 2.76]	2.70 [2.59, 2.81]	2.26 [2.20, 2.32]	2.57 [2.50, 2.65]	27.494	**<0.001**	P3 < P1, 2, 4; all ***p* < 0.001**
Death avoidance	2.84 [2.73, 2.94]	2.82 [2.70, 2.94]	2.66 [2.59, 2.73]	2.76 [2.68, 2.84]	3.723	**0.011**	P3 < P1, ***p* = 0.028**
Neutral acceptance	4.05 [3.98, 4.13]	3.87 [3.78, 3.95]	4.34 [4.30, 4.39]	4.12 [4.07, 4.18]	39.899	**<0.001**	P3 > P1, 2, 4; all ***p* < 0.001**; P1 > P2, ***p* = 0.005**; P4 > P2, ***p* < 0.001**
Approach acceptance	2.70 [2.61, 2.79]	2.74 [2.63, 2.84]	2.36 [2.31, 2.42]	2.62 [2.55, 2.69]	22.156	**<0.001**	P3 < P1, 2, 4; all ***p* < 0.001**
Escape acceptance	2.56 [2.45, 2.67]	2.65 [2.52, 2.77]	1.96 [1.89, 2.03]	2.30 [2.22, 2.38]	46.666	**<0.001**	P3 < P1, 2, 4; all ***p* < 0.001**; P4 < P1, ***p* = 0.001**; P4 < P2, ***p* < 0.001**

*Post hoc* pairwise comparisons were Bonferroni-corrected; only significant comparisons are shown. P1, Profile 1; P2, Profile 2; P3, Profile 3; P4, Profile 4. Bold values indicate statistical significance (*p* < 0.05).

After adjustment for education level, significant profile differences were observed for fear of death (*F* = 27.494, *p* < 0.001), death avoidance (*F* = 3.723, *p* = 0.011), neutral acceptance (*F* = 39.899, *p* < 0.001), approach acceptance (*F* = 22.156, *p* < 0.001), and escape acceptance (*F* = 46.666, *p* < 0.001; Table 6). In Bonferroni-corrected pairwise comparisons, Profile 3 showed lower adjusted means for fear of death, approach acceptance, and escape acceptance than Profiles 1, 2, and 4 (all *p* < 0.001), and the highest adjusted mean for neutral acceptance compared with Profiles 1, 2, and 4 (all *p* < 0.001); additionally, Profile 1 showed a significantly higher adjusted mean for neutral acceptance than Profile 2 (*p* = 0.005), and Profile 4 also showed a significantly higher adjusted mean than Profile 2 (*p* < 0.001). For death avoidance, only the difference between Profile 1 and Profile 3 was significant, with Profile 3 showing a lower adjusted mean (*p* = 0.028). In addition, Profile 4 showed lower escape acceptance than Profiles 1 and 2 (*p* = 0.001 and *p* < 0.001, respectively).

## 4 Discussion

### 4.1 Principal findings

This study employed a latent profile analysis to explore the heterogeneous nature of adult attachment among intern nursing students in China and its associations with death attitudes and intention to remain in nursing. Our results identified four adult attachment profiles among intern nursing students: relatively secure, mildly insecure, moderately insecure, and highly insecure. This finding aligns with person-centered studies in other populations, which also typically identify a secure group alongside multiple insecure groups characterized by different configurations of attachment anxiety and avoidance (28).

A gradient was observed in the associations between these profiles and death attitudes. Specifically, interns in the highly and moderately insecure profiles reported significantly higher levels of fear of death, death avoidance, and escape acceptance, and lower levels of neutral acceptance compared with their relatively secure counterparts. Notably, approach acceptance also differed across profiles; the relatively secure profile reported the lowest approach acceptance, which may reflect weaker endorsement of afterlife-based meanings of death rather than “worse” adjustment.

These findings are consistent with prior evidence linking attachment insecurity to more negative, avoidant, or escape-oriented attitudes toward death (15). Furthermore, the highly insecure profile showed the lowest proportion of interns intending to remain in nursing, whereas the highest intentions were observed in the relatively secure and mildly insecure profiles. These results suggest that higher attachment insecurity was associated with lower intention to remain in nursing, while mild insecurity did not necessarily preclude career commitment, indicating a nuanced relationship between attachment patterns and professional choices (20). One plausible explanation is that highly insecure interns may experience greater emotional dysregulation and interpersonal threat sensitivity during clinical encounters, which may make repeated exposure to death and patient suffering feel more overwhelming. By contrast, mildly insecure interns may retain sufficient relational resources and coping capacity to manage stressors and sustain motivation despite some insecurity. In end-of-life contexts, elevated death anxiety and avoidance may be related to greater distress and professional disengagement; whether these processes mediate the association between attachment insecurity and intention to remain in nursing could not be determined from the present cross-sectional data and warrants longitudinal investigation.

### 4.2 Structure and distribution of attachment profiles

Regarding the structural nature of these subgroups, the identified profiles in this study primarily reflect a gradient of attachment insecurity severity rather than aligning perfectly with the classic four-category model (secure, fearful, preoccupied, dismissing). This pattern is consistent with a growing body of LPA research that uses dimensional measures of attachment anxiety and avoidance, which often yields profiles characterized by levels of security/insecurity rather than pure categorical types (29). For instance, similar studies have identified a “highly secure” group with very low anxiety and avoidance, a “highly insecure” group with high scores on both dimensions, and one or two moderately insecure groups (30, 31). Our findings of a substantial relatively secure profile (41.6%) coexisting with a notable highly insecure profile (12.5%) indicate that a significant proportion of intern nursing students may enter the late stages of clinical training with considerable attachment insecurities. From an attachment-theoretical perspective, greater attachment security may be associated with more effective emotion regulation and more balanced threat appraisal in high-stress contexts, which may be associated with lower death-related fear, avoidance, and escape-oriented attitudes. By contrast, elevated attachment anxiety is typically linked to hyperactivation of the attachment system (heightened threat sensitivity and emotional arousal), whereas elevated attachment avoidance reflects deactivating strategies (emotional distancing and suppression); these mechanisms may help explain why profiles with greater insecurity tended to exhibit more negative, avoidant, or escape-oriented death attitudes.

As a tentative contextual interpretation, the one-child policy generation, which constitutes a significant part of the current nursing student population, might have experienced heightened parental expectations and overprotection, potentially fostering anxious or dependent relational patterns that translate into higher attachment anxiety in adulthood (32). Furthermore, the collective nature of Chinese society, while providing support, may also emphasize conformity and harmony, potentially leading some individuals to suppress autonomous needs (reflected in avoidance) or excessively seek approval (reflected in anxiety) in interpersonal relationships, including those in high-stakes clinical environments. As these cultural factors were not directly measured in the present study, these interpretations should be treated with caution and be tested directly in future research.

### 4.3 Attachment insecurity and death attitudes

Our findings suggested a graded association between attachment insecurity and death attitudes. As insecurity increased across profiles, interns reported higher fear of death, greater death avoidance, and stronger escape acceptance, alongside lower neutral acceptance. In contrast, the relatively secure profile showed the lowest fear and avoidance and the highest neutral acceptance. However, the differences in death avoidance across these attachment profiles were much smaller than those observed for other dimensions of death attitudes. This pattern suggests that avoiding death-related thoughts may be a broadly adopted coping mechanism among nursing interns, perhaps reinforced by cultural taboos and the emotional demands of clinical training, making it less contingent on individual attachment style. Even relatively secure interns might engage in some degree of death avoidance as a protective strategy, which could narrow the gap in avoidance behaviors between secure and insecure groups.

This pattern aligns with attachment theory, which suggests that internal working models shape how individuals appraise and cope with threats (33). For interns with high attachment insecurity, the existential threat of death may be experienced as more overwhelming and accompanied by intense anxiety (anxiety dimension) and/or a tendency toward psychological distancing from the stressor (avoidance dimension). In this context, death may be perceived as something to be feared, avoided, or seen as an escape from suffering, rather than an accepted part of life.

In contrast, secure individuals, equipped with positive models of self and others, may have greater psychological resources for approaching death-related experiences with less distress and a more balanced perspective (34). Clinically, these findings suggest that interns in the highly and moderately insecure profiles may be more vulnerable to experiencing intense anxiety and utilizing maladaptive coping mechanisms when facing dying patients, which may have implications for their wellbeing and engagement in end-of-life care.

These findings may inform efforts to move beyond one-size-fits-all approaches to death education and intern support. Attachment-informed screening, targeted psychological support, emotion regulation skills training, and reflective supervision are potential avenues worth exploring, and may help interns develop more adaptive attitudes toward death and support their professional development in palliative care contexts (35).

### 4.4 Escape acceptance as a marker of vulnerability

A particularly notable pattern in our findings is the pronounced gradient observed in escape acceptance, which was highest in the highly insecure profile (Profile 2), followed by the moderately insecure profile (Profile 1). Escape acceptance—a tendency to view death as a release from overwhelming psychological distress—has been strongly linked in prior research to maladaptive coping, emotional suppression, and chronic stress overload (36). The attachment configurations characterizing Profile 1 and Profile 2 may offer a tentative explanation for this pattern. Interns in the highly insecure profile showed simultaneously elevated attachment anxiety and avoidance, a combination associated with both hyperactivation and deactivation of the attachment system. The anxious component may heighten sensitivity to threat and intensify emotional arousal when facing death-related cues, whereas the avoidant component may promote emotional distancing and disengagement from distressing situations. When these conflicting tendencies coexist, individuals may experience unmanageable internal distress but lack effective strategies for processing or regulating these emotions, making escape-oriented appraisals of death particularly salient (37).

Similarly, those in the moderately insecure profile show elevated attachment anxiety, which may be associated with a greater tendency to view death as overwhelming and uncontrollable. Without sufficient relational security or coping resources, they may be more likely to endorse escape acceptance as a cognitive mechanism to reduce perceived helplessness.

In contrast, individuals in the relatively secure and mildly insecure profiles showed greater tolerance for emotional discomfort and more adaptive coping strategies, which may be associated with a lower tendency toward interpreting death as an escape. These mechanisms may help explain why escape acceptance showed the steepest gradient across profiles and highlight its potential role as a clinically meaningful marker of vulnerability among interns with higher attachment insecurity (38, 39).

It is also noteworthy that, beyond attachment-related factors, interns’ education level showed a significant association with escape acceptance in our results. This pattern may suggest that additional education and training may equip students with better coping skills and perspectives, and with a lower inclination to view death primarily as an escape from suffering. For example, longer study duration and greater clinical exposure in baccalaureate and graduate programs may provide more opportunities to encounter palliative care content and develop emotional resilience, potentially fostering more nuanced attitudes toward death and suffering (40). Conversely, students with fewer educational experiences in this area might feel less prepared to alleviate patients’ suffering, potentially being associated with a greater tendency to view death as a relief from pain. Consistent with this interpretation, prior research has noted that a higher academic level correlates with more accepting and less escape-oriented attitudes toward death among nursing students (9). Although the influence of education level was modest compared with attachment profiles, these findings suggest that formal training and experience may play a contributory role in how interns appraise death, complementing personal attachment factors in their coping repertoire.

### 4.5 Implications for intention to remain in nursing

Beyond psychological attributes, the results highlight the association between attachment insecurity and intention to remain in nursing. The significant divergence in intention to remain in nursing across the attachment profiles suggests that interpersonal security may be associated with students’ stated career intentions. Our findings indicate that interns in the highly insecure profile were the least likely to intend to remain in nursing, whereas those in the relatively secure and mildly insecure profiles showed the highest proportions intending to remain, with the moderately insecure group falling in between.

This pattern may be understood in relation to the chronic difficulties associated with insecure attachment in managing interpersonal and emotional stressors. Individuals with high attachment insecurity, characterized by hyperactivated anxiety or deactivated avoidance, may perceive the nursing environment as particularly stressful, which has been associated with emotional exhaustion and burnout (41). The core nursing tasks of building therapeutic relationships and managing intense patient emotions may be especially demanding for those with insecure internal working models. Furthermore, they may hold less favorable expectations about their efficacy in such an interpersonally demanding profession, which may be associated with lower confidence and reduced intention to remain in the field (42). Although causal conclusions cannot be drawn from the cross-sectional design, less favorable death attitudes, such as higher fear of death, death avoidance, and escape acceptance, may coexist with lower intention to remain in nursing among interns with higher attachment insecurity. However, because of the cross-sectional design, the temporal ordering and possible mediating relationships among these variables could not be determined. Future longitudinal research is warranted to examine whether death attitudes help explain the association between attachment profiles and intention to remain in nursing (15).

An important but often overlooked finding is the distinct position of the mildly insecure profile (Profile 4). Although this group exhibited intermediate levels of attachment anxiety and avoidance, their proportion intending to remain in nursing was almost as high as that of the relatively secure profile. This suggests that mild insecurity does not necessarily correspond to lower stated career intention. Instead, interns in this group may retain sufficient relational resources and regulatory capacities to manage clinical stressors despite some residual insecurity. From an educational perspective, this pattern suggests that attachment-informed support in nursing education may focus on identifying students with higher insecurity and providing appropriate support, rather than assuming that all forms of insecurity are equally associated with lower intention to remain. The mildly insecure group may therefore represent a subgroup that could benefit from supportive supervision, reflective training, and relational skills development, although this possibility requires testing in future intervention studies.

## 5 Implications

This study, by identifying distinct adult attachment profiles among Chinese intern nursing students and examining their associations with death attitudes and intention to remain in nursing, may inform the development of targeted support strategies. The following practice-oriented directions are tentatively proposed, with the caveat that their effectiveness requires empirical testing in future intervention studies. First, nursing schools and hospitals could consider exploring early screening for attachment insecurity and extreme death anxiety during the pre-internship or initial clinical placement phase, which may facilitate earlier identification of students who may benefit from additional support. Second, death education curricula may benefit from moving beyond theoretical knowledge. Integrating modules on emotion regulation skills, fostering relational security, and establishing regular reflective supervision or peer support groups could potentially support insecurely attached students in processing their clinical experiences. Third, clinical instructors and on-site psychological counselors may benefit from being trained to recognize signs of profound death avoidance or intense emotional reactions among interns. For these individuals, more intensive, individualized support may be considered. Finally, an attachment-informed perspective may help educators consider individual differences when designing death education and psychological support. Such approaches should be evaluated in future intervention studies before conclusions can be drawn about their effects on death attitudes or intention to remain in nursing. Collectively, these directions may contribute to a more responsive clinical learning environment that supports interns’ well-being; however, their practical effectiveness should be evaluated through rigorous future research.

## 6 Limitations and further research

Several limitations should be considered. First, the cross-sectional design precludes causal inferences regarding directionality or potential mediating relationships among attachment profiles, death attitudes, and intention to remain in nursing. Second, the use of convenience sampling from a single city (Hangzhou) limits the generalizability of the findings. Third, all data were self-reported, which may introduce social desirability bias and common method variance. Fourth, although we used validated instruments, the LPA relied on only two attachment indicators (avoidance and anxiety). Intention to remain in nursing was assessed using a single-item, dichotomous (yes/no) measure. Although similar approaches have been widely used in prior research, this format may not fully capture the multidimensional nature and gradations of intention to remain in nursing, thereby limiting measurement precision. Therefore, future studies are encouraged to employ validated multi-item instruments to provide a more comprehensive assessment of this construct. Furthermore, the use of only two continuous indicators raises the question of whether LPA offered substantive advantages over continuous dimensional modeling; this warrants direct comparison in future studies. Fifth, education level was included as a covariate given its conceptual relevance and significant variation across profiles. However, other potentially relevant factors, such as clinical exposure to death, prior bereavement experiences, and psychological characteristics (e.g., stress or resilience), were not measured. The omission of these variables may introduce residual confounding and could have influenced the observed associations between attachment profiles, death attitudes, and intention to remain in nursing. Therefore, future research is needed to incorporate a broader range of theoretically relevant covariates to better clarify these relationships. Sixth, although the five- and six-profile LPA models demonstrated slightly better information-criterion values, they exhibited unstable solutions (non-replicated loglikelihoods) and conceptually indistinct profiles. Therefore, the final four-profile solution should be interpreted with caution, and future work using larger samples or additional attachment indicators may help clarify whether more granular profiles are substantively meaningful.

Future research should employ longitudinal or experimental designs to clarify temporal ordering and examine possible explanatory pathways. Multi-center sampling across diverse regions in China is needed to enhance representativeness. Utilizing more comprehensive measures, including multi-item scales for retention-related outcomes and potentially incorporating behavioral or observational data, would strengthen the robustness of future findings.

## 7 Conclusion

In conclusion, this study provides a person-centered perspective on the psychological landscape of intern nursing students in China by identifying four distinct adult attachment profiles. The observed gradients across these profiles in relation to death attitudes and intention to remain in nursing suggest that attachment insecurity may be associated with less favorable death-attitude patterns and lower stated intention to remain in nursing. Specifically, higher attachment insecurity was associated with higher fear of death, death avoidance, and escape acceptance, as well as lower neutral acceptance and lower intention to remain in nursing. These findings may inform efforts to develop more tailored approaches to death education and psychological support in nursing training. Findings should be interpreted in light of the cross-sectional design, convenience sampling, self-report data, and single-item outcome measure. Longitudinal research is needed to clarify directionality and evaluate whether attachment-informed educational support can improve death-related coping and retention-related outcomes.

## Data Availability

The datasets used and/or analyzed in the current study can be made available by the corresponding author upon reasonable request.

## Footnotes

1
www.wjx.cn
