# Supplementary material for: Adult attachment profiles, death attitudes, and intention to remain in nursing among Chinese intern nursing students
Source: Front Med (Lausanne). 2026 Jun 29;13:1803579. doi: 10.3389/fmed.2026.1803579 (PMC13357581; doi:10.3389/fmed.2026.1803579)
Supplement: Supplementary file 1 [file Table_1.docx]

**Supplementary Table 1. Comparison of adult attachment, death attitudes, and intention to remain in nursing across education levels (*N* = 1,124)**

| **Variable** | **Associate degree**  **(n = 52) M ± SD** | **Bachelor degree**  **(n = 978) M ± SD** | **Master degree**  **(n = 94) M ± SD** | **F** | **p** | **Post hoc (Bonferroni)** |
| --- | --- | --- | --- | --- | --- | --- |
| AAQ-10 |  |  |  |  |  |  |
| Attachment avoidance | 10.52 ± 5.64 | 10.09 ± 5.02 | 9.01 ± 4.88 | 2.246 | 0.106 | ns |
| Attachment anxiety | 14.98 ± 6.91 | 15.86 ± 6.86 | 13.43 ± 7.00 | 5.602 | 0.004 | B > M, p = 0.003 |
| DAP-R |  |  |  |  |  |  |
| Fear of death | 2.45 ± 0.70 | 2.49 ± 0.69 | 2.28 ± 0.77 | 4.149 | 0.016 | B > M, p = 0.013 |
| Death avoidance | 2.85 ± 0.84 | 2.74 ± 0.72 | 2.66 ± 0.77 | 1.108 | 0.330 | ns |
| Neutral acceptance | 4.14 ± 0.52 | 4.16 ± 0.53 | 4.31 ± 0.53 | 3.499 | 0.031 | M > B, p = 0.027 |
| Approach acceptance | 2.64 ± 0.66 | 2.55 ± 0.64 | 2.37 ± 0.75 | 3.994 | 0.019 | A > M, p = 0.049; B > M, p = 0.029 |
| Escape acceptance | 2.32 ± 0.93 | 2.26 ± 0.79 | 2.02 ± 0.85 | 4.072 | 0.017 | B > M, p = 0.017 |
| Intention to remain, n (%) | 39 (75.0%) | 767 (78.4%) | 83 (88.3%) | χ² = 5.607 | 0.061 | ns |

***Note.*** A = Associate degree; B = Bachelor degree; M = Master degree. One-way ANOVA with Bonferroni-corrected post hoc comparisons was used for continuous variables. Chi-square test was used for intention to remain in nursing. ns = not significant.
